# Supplementary material for: Coalescing beneficial host and deleterious antiparasitic actions as an antischistosomal strategy
Source: eLife. 2018 Jul 30;7:e35755. doi: 10.7554/eLife.35755 (PMC6095690; doi:10.7554/eLife.35755)
Supplement: Transparent reporting form [file elife-35755-transrepform.docx]

***eLife’s* transparent reporting form**

We encourage authors to provide detailed information *within their submission* to facilitate the interpretation and replication of experiments. Authors can upload supporting documentation to indicate the use of appropriate reporting guidelines for health-related research (see [EQUATOR Network](http://www.equator-network.org/%20)), life science research (see the [BioSharing Information Resource](https://biosharing.org/)), or the [ARRIVE guidelines](http://www.plosbiology.org/article/info:doi/10.1371/journal.pbio.1000412) for reporting work involving animal research. Where applicable, authors should refer to any relevant reporting standards documents in this form.

If you have any questions, please consult our Journal Policies and/or contact us: [editorial@elifesciences.org](mailto:editorial@elifesciences.org).

**Sample-size estimation**

- You should state whether an appropriate sample size was computed when the study was being designed
- You should state the statistical method of sample size computation and any required assumptions
- If no explicit power analysis was used, you should describe how you decided what sample (replicate) size (number) to use

Please outline where this information can be found within the submission (e.g., sections or figure legends), or explain why this information doesn’t apply to your submission:

Sample size calculations, including thresholds for significance criterion and power, for all animal work is outlined in the Statistics paragraph of the Materials and Methods section of the manuscript, specifically justifying animal sample sizes in Figure 6A&B.

**Replicates**

- You should report how often each experiment was performed
- You should include a definition of biological versus technical replication
- The data obtained should be provided and sufficient information should be provided to indicate the number of independent biological and/or technical replicates
- If you encountered any outliers, you should describe how these were handled
- Criteria for exclusion/inclusion of data should be clearly stated
- High-throughput sequence data should be uploaded before submission, with a private link for reviewers provided (these are available from both GEO and ArrayExpress)

Please outline where this information can be found within the submission (e.g., sections or figure legends), or explain why this information doesn’t apply to your submission:

*In vitro* cell based screens: Primary high throughput screen was performed as a single replicate, as stated in the results section of the manuscript. All data from the primary screen is also provided in Sheet 1 of Supplemental Table 1. Hit validation and counter screening to triage off target results was performed in biological triplicates, each consisting of technical quadruplicates, as stated in the “cAMP luminescence assays” section of the Materials and Methods. Dose response curves for ergot alkaloid structure activity screening reflect values from biological triplicates, as stated in the Figure 3 legend.

*Ex vivo* parasite screens: Worm movement dose response curves and egg laying assays were performed in biological triplicates, as stated in the legends of Figure 4 and Supplemental Figure 4 and the “*Ex vivo* schistosome assays” section of the Materials and Methods.

*In vivo* assays: Hepatic shift assays were performed in biological triplicates, as stated in the “*In vivo* schistosome drug screening” section of the Materials and Methods. Numbers of mice used in survival analysis are stated in the legend of Figure 6. Raw data for individual data points representing mouse worm burdens in control and drug treated infections are shown in Figure 6B (DMSO control = 20 mice, ERG = 14 mice, PZQ = 8 mice).

RNASeq of mouse organs: Each cohort consisted of five biological replicates, as illustrated in the experimental schematic shown in Figure 7A and the legend of Figure 7.

Biological replication is defined as cohorts of independent animal samples, or in the case of *in vitro* cell based assays runs of cultured cells independently plated in separate plates/wells and assayed for drug responsiveness. No outliers were excluded, therefore no criteria for exclusion/inclusion of data was used.

All raw RNASeq data for each biological sample (20 mice in total) from Figure 7 has been archived at NCBI SRA database under accession number SRP131511, and mapped read counts for each replicate are shown in Supplemental Tables 2&3.

**Statistical reporting**

- Statistical analysis methods should be described and justified
- Raw data should be presented in figures whenever informative to do so (typically when N per group is less than 10)
- For each experiment, you should identify the statistical tests used, exact values of N, definitions of center, methods of multiple test correction, and dispersion and precision measures (e.g., mean, median, SD, SEM, confidence intervals; and, for the major substantive results, a measure of effect size (e.g., Pearson's r, Cohen's d)
- Report exact p-values wherever possible alongside the summary statistics and 95% confidence intervals. These should be reported for all key questions and not only when the p-value is less than 0.05.

Please outline where this information can be found within the submission (e.g., sections or figure legends), or explain why this information doesn’t apply to your submission:

*In vitro* cell based screens: Metrics for evaluating Sm.5HTRL cAMP luciferase assay performance are stated in the “Statistics” section of the Materials and Methods and elaborated on in Figure 1-figure supplement 3, where all equations are provided. Raw data used to generate Figure 2 is provided in Supplemental Table 1. Each data point in the dose response curves presented reflects the mean +/- standard error, as stated in legend of Figure 3.

*In vivo* schistosome assays: Data in Figure 6B,C,E&F are shown as unpaired t-tests comparing drug treated sample to DMSO controls. The log-rank test was used to compare survival of ergotamine and DMSO treated infected animals. This detail, as well as the exact p value and hazard ratio are stated in the results section of the manuscript.

Differentially expressed gene products in mouse livers and spleens were determined using EdgeR generated, Benjamini-Hochberg adjusted p values. Adjusted p values < 0.05 were considered statistically significant. The exact p values for each gene product are provided in Supplemental Tables 2 & 3.

Ingenuity pathway analysis of differentially expressed gene products utilizes Fisher exact test, as stated in the “Statistics” section of the Materials and Methods and the Results section of the manuscript. Exact p values for pathways analyzed and gene products assigned to each are provided in Supplemental File 4.

(For large datasets, or papers with a very large number of statistical tests, you may upload a single table file with tests, Ns, etc., with reference to sections in the manuscript.)

**Group allocation**

- Indicate how samples were allocated into experimental groups (in the case of clinical studies, please specify allocation to treatment method); if randomization was used, please also state if restricted randomization was applied
- Indicate if masking was used during group allocation, data collection and/or data analysis

Please outline where this information can be found within the submission (e.g., sections or figure legends), or explain why this information doesn’t apply to your submission:

Cages of mice were randomly selected to be treated with either DMSO control, PZQ or ERG. Restricted randomization was not applied. No masking was used in group allocation or data collection/analysis.

**Additional data files (“source data”)**

- We encourage you to upload relevant additional data files, such as numerical data that are represented as a graph in a figure, or as a summary table
- Where provided, these should be in the most useful format, and they can be uploaded as “Source data” files linked to a main figure or table
- Include model definition files including the full list of parameters used
- Include code used for data analysis (e.g., R, MatLab)
- Avoid stating that data files are “available upon request”

Please indicate the figures or tables for which source data files have been provided:

Numerical values for drug screening data in Figure 2 are provided in Supplemental File 1.

All read mapping data used to generate Figure 7 has been provided in Supplemental Files 2 & 3. FASTQ files of raw sequencing data has also been deposited in the NCBI SRA database under accession number SRP131511.
